# Herbal Medicine Uses for Respiratory System Disorders and Possible Trends in New Herbal Medicinal Recipes during COVID-19 in Pasvalys District, Lithuania

**DOI:** 10.3390/ijerph19158905

**Published:** 2022-07-22

**Authors:** Zivile Pranskuniene, Ruta Balciunaite, Zenona Simaitiene, Jurga Bernatoniene

**Affiliations:** 1 Department of Drug Technology and Social Pharmacy, Lithuanian University of Health Sciences, Sukileliu pr. 13, LT-50162 Kaunas, Lithuania; jurga.bernatoniene@lsmuni.lt; 2 Institute of Pharmaceutical Technologies, Lithuanian University of Health Sciences, Sukileliu pr. 13, LT-50162 Kaunas, Lithuania; ruta.balciunaite@stud.lsmu.lt; 3 The Museum of History of Lithuanian Medicine and Pharmacy, Rotušės 28, LT-01100 Kaunas, Lithuania; zenona.simaitiene@lsmu.lt

**Keywords:** herbal medicine, COVID-19, respiratory system disorders, Lithuania

## Abstract

Despite some preliminary studies of the available herbal medicine preparations and their curative effects on COVID-19, experts still fear that unproper use of such homemade medicines could do more harm than good to people relying on unproven alternatives of questionable efficacy. The main purpose of this study was to evaluate the safety of herbal medicines used for respiratory system disorders in the Pasvalys district during the COVID-19 pandemic in Lithuania. An archival source was also studied, looking for possible recipes for the treatment and prevention of respiratory diseases in Lithuanian traditional medicine, emphasizing the safety guidelines. The survey was conducted using the deep interview method. The respondents mentioned 60 species of medicinal plants from 29 different families used for the treatment and prevention of respiratory system disorders (for cough mostly, 51.70% of all indications). Twenty eight out of 60 plant species were not included in the European Medicines Agency monographs and only 50% of all included species were used as indicated by the European Medicines Agency for respiratory system disorders. The trends in the ethnopharmacological choices of modern consumers and the analysis of archival sources can be a great source of ideas for new herbal-based pharmaceutical preparations for COVID-19 symptoms in Lithuania considering the safety recommendations.

## 1. Introduction

Traditional medicine is a set of knowledge, skills, and practices based on the theories, beliefs, and experiences of different cultures used to improve health by preventing and treating physical and mental disorders [1]. Folk medicine includes home-made medicines, rituals, and procedures, forms of treatment used by the self-taught to alleviate the symptoms of diseases, and for disease prevention [2]. Before the modern medical system was developed, folk medicine was the only way to combat various diseases and ailments, but a large part of the world’s population still uses traditional and folk treatments [1,3,4]. Most countries aiming to preserve their cultural history nurture and preserve the knowledge of folk medicine, passing on the accumulated knowledge from generation to generation [1].

The traditional knowledge associated with medicinal plants nowadays is gaining importance worldwide, due also to the emergence of the new disease of COVID-19 [5]. In the winter of 2019, a new SARS-CoV-2 virus was detected in patients with pneumonia in Wuhan, China. COVID-19 is an infectious disease caused by the SARS-CoV-2 virus. Most people infected with the virus will experience mild to moderate respiratory illness and recover without requiring special treatment, but some will become seriously ill and require intensive medicinal care.

The COVID-19 pandemic has become the greatest threat to humanity in the 21st century, and its impact on the health and economy is expected to be even more severe for communities located in remote areas [6,7]. The situation underlines the need for ethnobotanists to adapt to the situation and complement the efforts of local communities and authorities in the fight against the COVID-19 pandemic. The relationship between disease prevalence and the medicinal plants, and the ability of local pharmacopoeias to respond to emerging diseases is a widely understood concept of ethnobiology [8,9]. Historical evidence evaluated through the 20th and 21st century suggests that during various pandemics, medicinal plants have played a role in treatment before the arrival of major vaccines [10].

Viral respiratory infections are some of the most common infections worldwide, with the majority of the world’s population suffering from at least one infection a year [6]. Respiratory diseases are known to affect the airways including the nasal cavity, bronchi, and lungs. Very often, it is difficult to classify these diseases because they vary in severity and are highly dependent on the age of the patient. Although most of the ethnobotanical research on plants potentially acting on the respiratory function and against COVID-19 symptoms has been performed in countries where primary health care is difficult to access [11,12], general upward trends in traditional medicine are also seen in developed countries [5]. According to ethnopharmaceutical research conducted thus far, respiratory diseases come first or second in Lithuania as the indication group for using ethnopharmacological preparations [13,14,15].

According to the general trend of ethnopharmacological measures used for COVID-19, the most popular are plants that have medicinal properties against influenza and malaria as well as plants with immunomodulatory properties, and plants that have a positive effect on respiratory disorders [7,10]. Medicinal plants have long been known for their therapeutic or protective effects against viruses, protozoa, bacteria, and parasites [16]. Cough is one of the most common symptoms experienced along with fever as well as shortness of breath, tiredness, and headache. Plants for the suppression of these symptoms may be beneficial to patients with COVID-19 [7].

The limited availability and accessibility of conventional medicine during COVID-19 have made traditional medicines more appealing due to their wide availability and increased position of safety. Several herbal medicines are believed to alleviate or cure COVID-19 and its symptoms [5]. Despite some preliminary study of the available herbal medicine preparations and their curative effects on COVID-19, experts still fear that improper use of such homemade medicines could do more harm than good to people relying on unproven alternatives of questionable efficacy [5,9].

Researchers of ethnopharmacology have noted that the global COVID-19 pandemic forced individualization in the home environment, and the collapse of the entire health care system made people turn back to the forgotten knowledge of traditional medicine to have both medicine and food ready at one’s fingertips [17]. Even if treatment of COVID-19 is based on modern medicine, the associated symptoms of COVID-19 and long-COVID are still newcomers in medicine, and ethnopharmacological measures are being sought for the long-term relief of symptoms. Often herbal medicine is chosen not only for its efficacy but also for its assumed safety, not considering that the long-term use of the same natural medicinal substances can have negative health consequences. Most plants used for therapeutic and prophylactic purposes during a COVID-19 pandemic may contain toxic substances that can cause a variety of health problems in the event of an overdose, making the research-based use important [18].

The main purpose of this study was to evaluate the safety of ethnopharmacology used to treat respiratory tract disorders in the Pasvalys district during the COVID-19 pandemic in Lithuania through the assessment of the compliance of the indications for the use of medicinal plants with European Union herbal monographs. The archival source of E. Šimkūnaitė (dated 1925–1947), with recipes of the famous herbalist in Lithuania, which became especially popular during the COVID-19 pandemic, was also evaluated. On one hand, ethnopharmaceuticals may be an excellent alternative to medication, especially during and after COVID-19, but special attention should be paid to safety recommendations. The therapeutic effects of herbal preparations are often underestimated, but the potential for overdose is also underestimated, especially when the nearest health care professional is not readily available. Critical and scientific study of the traditional treatment for COVID-19 may also act as the basis of counternarrative and education in response to the growing body of misinformation surrounding alternative COVID-19 treatment [5]. In addition, the analysis of archival sources can help to find possible trends in new herbal medicine recipes for and after COVID-19, based on traditional Lithuanian medicine. In many countries, there is growing interest in local traditional knowledge, leading to the creation and scientific analysis of recipes. This is the first study in Lithuania during the COVID-19 period to investigate the ethnopharmaceuticals for the prevention and treatment of respiratory diseases. A study of an archival source was also carried out, looking for possible recipes for the treatment and prevention of respiratory diseases in Lithuanian traditional medicine, emphasizing the guidelines for safe use.

## 2. Materials and Methods

### 2.1. Study Area

**Figure 1:**
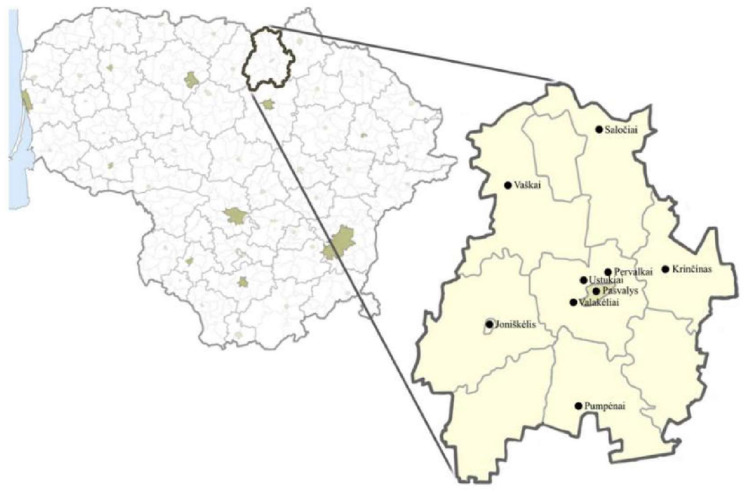
The study area.

The study was conducted in the Pasvalys district located in the northern part of Lithuania, in Panevėžys county, near the Latvian border (Figure 1). This is the area of the northern plains (Žemgala, Mūša-Nemunėlis lowlands) and fertile river valleys [19].

The district has no lakes, and there are only a few ponds. Karstic depressions, sinkholes, are characteristic of the Pasvalys district. Forests cover 16.6% of the municipal area, with deciduous forests the most common.

Pasvalys is located at the confluence of Lėvuo and Svalia. The name of the town was first mentioned at the end of the 13th century. In 1497, the Grand Duke of Lithuania Alexander donated land to the church started by the priest J. Grotas, and allowed the town to be established. Pasvalys was developed along the main road to Riga, so the town suddenly became a center of merchants and craftsmen [20]. In Pasvalys, at the end of the 19th century, mineral waters were used to treat patients [19].

The area of the Pasvalys district is 1289 km^2^, which makes up 2% of the total area of Lithuania. According to the data provided in 2020, there were 22,816 inhabitants in the Pasvalys district. There are two big towns in the district, Pasvalys and Joniškėlis; seven smaller towns, Daujėnai, Krikliniai, Krinčinas, Vaškai, Pumpėnai, Pušalotas, and Saločiai; and 398 villages [21]. Most of the population lives in villages and towns, which is why people tend to be treated with natural substances for mild diseases. Figure 1 shows the territory of the Pasvalys district, and the black circles indicate locations where the survey was conducted.

### 2.2. Methods

#### Research Organization, Respondents, and Methods

The study was conducted in 2021, from May to October in the Pasvalys district, Lithuania (Figure 1). During this period, 30 respondents (28 women and two men) living in the villages of Pervalkai, Ustukiai, and Valakėliai; the small towns of Krinčinas, Pumpėnai, Saločiai, Vaškai; and the towns of Pasvalys and Joniškėlis were interviewed. The study was conducted in accordance with the Code of Ethics of the International Society of Ethnobiology [22]. The study was approved by the Bioethics Center of the Lithuanian University of Health Sciences (No. BEC-FF-80).

To begin with, we aimed to become acquainted with and gather information about the people living in the study area. All participants in the study not only treated and prepared herbal pharmaceutical forms themselves, but also advised and taught how to prepare them to others who sought help. This target group was made up of herbalists, but only one respondent in this group sold prepared pharmaceutical products and accepted money for the consultation of how to overcome health problems with natural remedies and incantations.

A qualitative “snowball” method was used to search for respondents, during which the interviewee suggested another person who could share knowledge about the use of natural substances for the treatment and prevention of respiratory diseases. The research method was a structured interview. The respondent was initially introduced to the topic of the study and given the summary information, then an informed consent form to participate in the study was signed. The interview was conducted using a ready-made questionnaire. The questionnaire consisted of 16 questions that included a structured part of the interview. With the help of the questionnaire, the demographic data of the respondents, information about the cultivated and collected medicinal plants, their parts, preparation methods, and indications were collected. The investigators marked the received information in their notes, audio recordings on the Dictaphone were made with the respondent’s consent, and all data were encrypted. Additionally, some respondents were visited for a second time to supplement and clarify the information. The study identified the indications for use by the respondents and compared them with the monographs presented in the European Medicines Agency (EMA) assessment [23]. The purpose of the comparison was to determine the percentage of information on the indications for use obtained in the study that were consistent with the indications approved in the EMA studies.

Taxonomic identification, botanical nomenclature, and plant family assignment were performed based on the validated databases of the World Flora Online database [24] and Angiosperm Phylogeny Group IV [25]. Plant species were identified using the inscriptions of the traditional Lithuanian flora [26,27,28].

The data obtained during the research were compared with the archival source of E. Šimkūnaitė (dated 1925–1947), kept at the Museum of the History of Medicine and Pharmacy of the Lithuanian University of Health Sciences, using the method of comparative analysis. Eugenija Šimkūnaitė was a famous pharmacist, herbalist, ethnographer, and habilitated doctor of the biological sciences. The woman devoted her entire life to the study of medicinal plants and folk medicine. This archival source was chosen due to the scientific structure, methodology, and identified plant species, and the increased popularity and availability of E. Šimkūnaitė’s recipes during the COVID-19 pandemic [2].

The research data are stored at the Museum of the History of Medicine and Pharmacy of the Lithuanian University of Health Sciences.

## 3. Results and Discussion

### 3.1. Demographic Characteristics of Respondents and Sources of Ethnobotanical Knowledge

Thirty respondents (28 women and 2 men) participated in the study, and all respondents agreed to provide data on the use of natural substances for respiratory disease treatment and prevention. The percentage of men and women was uneven, because traditionally, knowledge of herbal medicine in Lithuania is transmitted through the women’s line and even nowadays, usually herbalists are women. Most respondents who participated in the structured interview were older adults and the elderly, 23 (84.40% of all respondents) belonged to the category of 50 years and older.

The study focused on the education of the respondents. Most respondents had secondary or special secondary education, 27% each. A significant part of the respondents (26%) had obtained a higher education. This was followed by respondents with higher vocational education (17%). The smallest part (3%) was the category of people with basic education. Most respondents had a secondary or vocational secondary education, those with secondary education did not acquire a specialty, and mostly indicated that they were housewives, and respondents who had a vocational secondary education most often indicated being a technician and an accountant. Respondents with a higher education indicated being a health care professional: nurses, veterinarians, and physiotherapists, but these were a minority. Considering the results of the research, we can state that few people among the respondents worked in health care; however, they all practiced traditional medicine and were basically self-taught.

**Figure 2:**
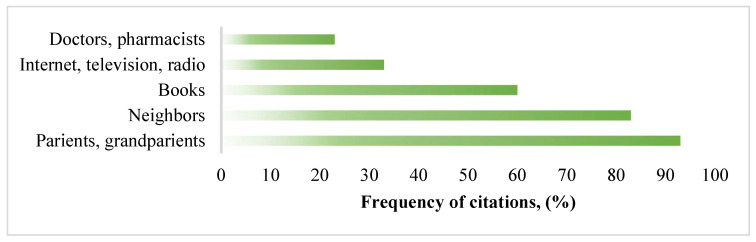
The ethnopharmacological information sources of the respondents.

It was very important to prove the authenticity/reliability of the knowledge gathered during the study. The structured interview asked respondents where they had gained the knowledge about traditional medicine, the use of medicinal plants, and how they had learned to treat with the help of natural substances. The results on the sources of information obtained during the study are presented in Figure 2.

Almost all respondents mentioned that they had acquired ethnopharmacological knowledge from their parents and grandparents, such answers accounted for as much as 93%. These results indicate that traditional medical knowledge is passed down from generation to generation to preserve knowledge about natural remedies. Most respondents stated that they had treated their children with folk remedies, who in turn now opted for natural treatments for their offspring. Such answers show that people tend to trust traditional medicine and it is not forgotten to this day. Furthermore, a large proportion of respondents cited neighbors and acquaintances as a source of information (83% of all citations). Thus, the respondents tended to share and exchange their accumulated knowledge of traditional medicine. Books and magazines as a source of information were cited by 60% of respondents, and 33% mentioned the radio, TV, and the Internet. It is interesting that traditional herbalists mentioned the Internet, TV, and radio in such a high percentage. This may also indicate that the respondents who were practitioners and actively interested in this field used all of the available information tools, and followed the current affairs and latest trends of traditional medicine through the radio, television, and even Internet. Only 23% consulted doctors and pharmacists to improve their knowledge on natural substances. The respondents stated that due to the epidemic situation, they were more likely to see a pharmacist than a doctor because pharmacists were more accessible during the COVID-19 pandemic. A pharmacist was seen by 63.30% of respondents and 23.30% contacted a doctor. Only a small part of the respondents answered that they did not turn to a health professional, and such answers accounted for 13.30% of the total citation frequency. However, when comparing the results of other ethnopharmaceutical research in Lithuania, we observed the opposite, where respondents did not turn to a doctor and/or pharmacist for ethnopharmaceutical knowledge and did not trust health care professionals in this matter [13,14]. The data from this study showed that respondents, even with ethnopharmaceutical knowledge, tended to consult health professionals (pharmacists), possibly influenced by the COVID-19 pandemic.

### 3.2. Indications for Use, the Most Popular Plants and Their Parts, Methods of Preparation

This ethnopharmaceutical study in the Pasvalys district identified 60 species of medicinal plants belonging to 29 families for the treatment and prevention of respiratory diseases. All of the collected data are summarized in detail in Table S1, and the analysis of archival sources in Table S2 (Supplementary Materials). Most often cited were *Thymus vulgaris* L. (22 citations), *Tilia cordata* Mill. (17), *Matricaria recutita* L. (19), *Rubus idaeus* L. (14), *Tussilago farfara* L. (eight citations) and *Foeniculum vulgare* Mill., *Glycyrrhiza glabra* L., *Salvia officinalis* L., *Mentha x piperita* L., and *Calendula officinalis* L. (six citations each). These herbs are popular for the treatment of upper respiratory tract diseases as other ethopharmaceutical research in Lithuania has also shown. During the first phase of the pandemic (December 2019–August 2020), the most frequently reported species during the pandemic named by the Lithuanians were *Allium sativum* L., *Allium cepa* L., *Urtica dioica* L., and *Tropaeolum majus* (L.) Kuntze [17]. In the Pasvalys study, these plant species were not so popular. Recently, a study in China indicated the *Radix glycyrrhizae* rhizome [29] as being well-known for its healing properties. According to the Chinese guidelines, it can be used for the treatment of COVID-19 patients; however, a clinical process is still to produce evidence [10].

**Figure 3:**
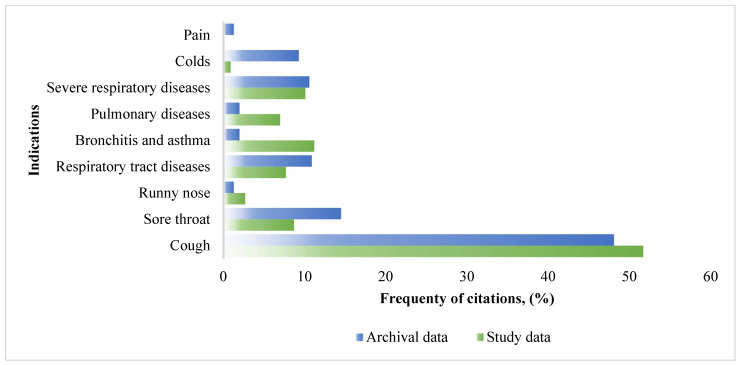
A comparison of the indications for use collected in ethnopharmacological research in the Pasvalys district and from the archival source. Severe respiratory diseases, pulmonary diseases, and respiratory tract diseases are the groups of diseases, named by respondents, but not specified.

The main indication for use was cough, as shown by the overall study data (Figure 3). From the data of the study, we can see that the respondents most often used herbs to relieve symptoms such as cough and shortness of breath (dry cough, severe cough attacks, shortness of breath), and such responses accounted for 51.70% of all citations. The use of herbs that relieve cough mostly intended to remove the secretion from the lungs and thus facilitate expectoration. In second place, subjects used herbs for bronchitis and asthma, accounting for 11.20% of all citations. Severe respiratory and organ diseases such as lung cancer, whooping cough (pertussis), various forms of tuberculosis, and angina accounted for 10.10% of all citations. Comparing the indications for use with the archival data, it was observed that cough and dyspnea were the main symptoms that subjects rushed to treat with the help of medicinal plants.

**Figure 4:**
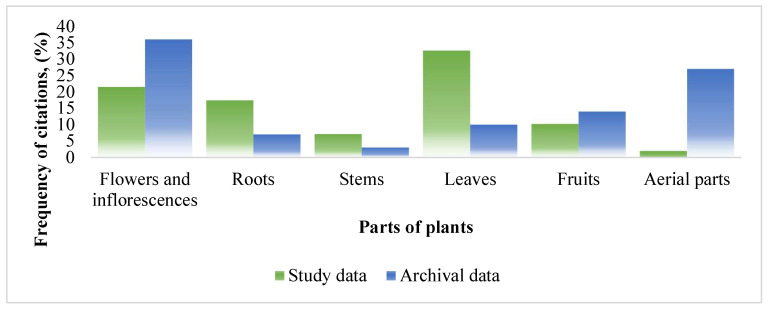
A comparison of the plant parts used in the ethnopharmacological study in the Pasvalys district and in the archival source.

Different parts of the plant (leaves, roots, flowers, or fruits) have different active ingredients, which can make one part of the plant toxic and the others beneficial to health. Phytotherapy may use the whole plant or only a part of the plant [30]. The parts of medicinal herbal raw materials and their use distribution are shown in Figure 4. According to the data of the study, we can see that leaves were the most frequently chosen part of the plan (32.60% of the citation frequency), while in the archival data, the leaves accounted for only 10% of the citation frequency. Flowers and inflorescences were very popular both in the study and in the archival data. In the study, they accounted for 21.50% of the citation frequency, and in the archival work, as much as 36% of the citation frequency. Respondents stated that leaves and flowers were the easiest to apply in traditional medicine. In the archival data, the whole plants were used very often (27% of the citation frequency), but in the study conducted in the Pasvalys district, the use of whole plants was not often mentioned (only 2% of the citation frequency). The roots accounted for 17.40% of the citation frequency in the Pasvalys study and 7% in the archives.

**Figure 5:**
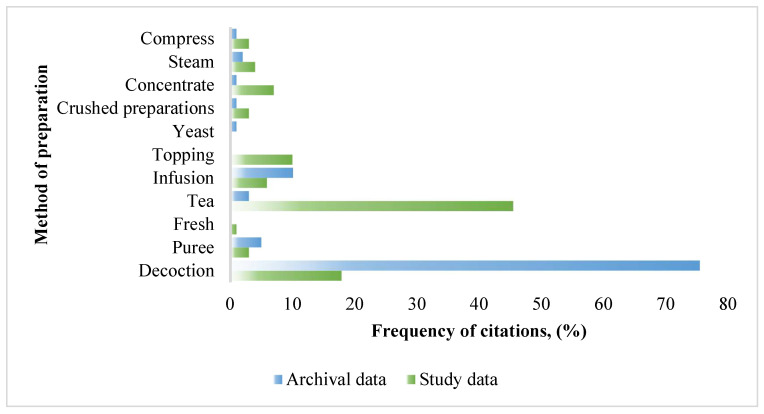
The methods of preparing the herbal medicines.

The aboveground parts of the plants were most often selected by the study participants and the most popular method of preparation was tea (Figure 5). While medicinal herbal teas are purposely consumed to treat a specific condition (i.e., cough) for a limited number of days, there is no limit to the duration for recreational teas as they are used within a food context, and not for the treatment of medical conditions [31,32]. Herbal beverages prepared as teas are consumed in a food context for their general social and/or recreational value or for their general attributions of being “healthy” drinks. In Eastern Europe, the most popular species was *Rubus idaeus* L. [33]; in our Pasvalys study, this trend was also confirmed. Herbs used as recreational teas, due to the Lithuanian climate, are also used to relieve colds and upper respiratory tract disorders and to strengthen their immunity [32]. Important sensory properties of recreational teas such as smell, flavor, and color must be suitable for making tea that has a tendency for long-term use. The long-term use of plants containing active compounds, especially without exchanging plant species, has raised some safety concerns [34]. This issue is of particular importance during the period of COVID-19 disease, when the use of home-made pharmaceutical products of plant origin, which themselves may cause adverse effects, is prolonged.

According to the research, medicinal plants are prepared as various pharmaceutical forms such as teas (herbs are added to boiling water), decoctions (herbs are boiled), and infusions (raw material is added to alcohol or vodka and left to extract), poultices (a cloth for poultice is soaked in decoction), and ointments (substances of plant origin and of animal origin are mixed). The preparation methods for the treatment of respiratory diseases and data from the archival sources are compared in Figure 5.

According to the data obtained in the Pasvalys district survey, the most popular method of preparation was tea (45.5% of the total citations), while in the archival data, the most frequently mentioned preparation method was decoction (75.5% of the total citations). In the archival data, teas accounted for only 3% of the total citation frequency, and in this study conducted in the Pasvalys district, decoction was mentioned in 17.9% of all citations. The decoctions were made with water or milk, sweetened with honey, and heated for a few minutes, while the tea was prepared simply by pouring boiling water over the herbs. This tendency to use a simple method of preparation (tea) only proves that medicinal plants were intended for long-term treatment of respiratory diseases, which would not require a more thorough preparation. According to the data of the archival analysis, the more thorough preparation method (decoction) with an increased concentration of active substances was intended for short-term treatment. Comparing the new Pasvalys survey to the historical data, it was observed that the preparation methods of medicinal plants among the modern respondents was poorer, simplified, and less attention was paid to the amounts of raw material, preparation time, and other details. The limited time of use and the exact amount of the herbal raw material were emphasized in the archival material, as was the necessity to consult a physician about its prolonged use [35].

### 3.3. Safety of Herbal Raw Materials and Possible Trends in New Herbal Medicinal Recipes

As the global use of herbal medicinal products continues to grow and many more new products are introduced into the market, public health issues and concerns surrounding their safety are also increasingly recognized [36]. Most plants used for therapeutic and prophylactic purposes during a COVID-19 pandemic may contain toxic substances (phenol, colchicine, etc.) that can cause a variety of health problems in the event of an overdose [37]. To evaluate the safety and efficacy of the investigated herbal medicines, we compared the results obtained in the Pasvalys district study and the data from the archival source with the recommendations provided by the EMA.

During interviews in the Pasvalys study, the adverse effects of herbs or herbal preparations were not recorded but were most likely not identified because the respondents underestimated the potential adverse effects of herbal medicine. The results obtained in the study conducted in the Pasvalys district showed that only 32 out of 60 herbal raw materials used by the respondents were described in the EMA monographs (Table S1). The other 28 were used in addition to the EMA recommendations for use. The indications identified in our study for the 32 herbal raw materials with the EMA assessment were 50% consistent with the EMA recommendations. The analysis of the archival data (Table S2) found that only 14 of the 69 herbal medicinal raw materials were described in the EMA monographs, and the remaining 55 did not have an EMA assessment. Seven from the 14 raw materials described (50%) had indications for use corresponding to the EMA recommendations (Table 1).

The most popular and commonly used medicinal plants of which the indications for use did not comply with the EMA recommendations were the seeds, leaves, and flowers of the burdock (*Arctium lappa* L.), which were used to treat cough and tonsillitis, the roots were used to treat tonsillitis, but the EMA recommends using roots for the treatment of the urinary tract, appetite improvement, and seborrheic skin diseases. Horsetail (*Equisetum arvense* L.) herbal tea was used by the subjects to relieve cough, but in an EMA monograph, horseradish is used to treat urinary tract and superficial wounds. Mint tea and infusions of mint (*Mentha x piperita* L.) were used by respondents to relieve cough and for inhalations for cough and blocked nose. According to the EMA recommendations, mint leaves are used to treat various digestive disorders (dyspepsia, bloating). *Urtica dioica* L. was frequently mentioned by the respondents and its leaves and roots were used to treat asthma and cough. According to the EMA recommendations, the leaves of this medicinal plant are used to relieve joint pain and as a supplement in mild urinary tract disorders, and the roots are used to relieve lower urinary tract symptoms associated with benign prostatic hyperplasia. St. John’s wort (*Hypericum perfoliatum* L.) was used for sore throat and cough, but according to the EMA recommendations, St. John’s wort should be used to reduce indigestion and relieve anxiety symptoms. Strawberry (*Fragaria vesca* L.) leaves were used by the respondents to treat cough, and the EMA guidelines use strawberry leaves for mild diarrhea and urinary tract treatment.

It is also important to mention that coltsfoot (*Tussilago farfara* L.) was mentioned many times by the respondents, and is listed among the most popular medicinal plants in the Pasvalys district, but according to the latest scientific literature, it is quite toxic when used internally. Researchers have found that coltsfoot alkaloids have a hepatotoxic effect on the liver [38]. Restrictions in the intake of pyrrolizidine-containing herbs and further investigations have been recommended because of the paucity of data on the toxicity in humans. The respondents used coltsfoot leaves and flowers to treat various diseases of the respiratory system: cough, asthma, lung diseases, and sore throats. The plant is widespread throughout the world and is widely used in folk medicine [38]. The EMA does not provide indications for the use of coltsfoot.

Despite not being approved by regulatory bodies as a definitive guideline in COVID-19 management yet, the application of ethnomedicine has resulted in several herbal medicine candidates that show a promising result regarding its efficacy in managing COVID-19 [5,39]. In many countries, traditional medicinal systems have tried to adapt to the new disease. For example, Ayurveda recommends preventing the disease progression by regulating the immune-inflammation state in COVID-19 patients. Decoctions of *Ocimum sanctum* L., *Piper nigrum* L., *Zingiber officinale* Roscoe, *Cinnamomum verum* J. Presl, and *Vitis vinifera* L. have been used to improve the immunity in COVID-19 patients. Patients with sore throat and cough are advised to carry out steam inhalation with *Mentha arvensis* L. with *Syzigium aromaticum* L. powder [40,41]. According to the Pasvalys study, inhalation with *Lavandula angustifolia* Mill., *Melissa officinalis* L., and *Mentha x piperita* L was used to relieve cough.

Several traditional Chinese medicine formulations used after the outbreak of COVID-19 have significant antiviral, anti-inflammatory, and immunomodulatory activity. Based on recent updates, several herbs and isolated phytomolecules have been found to inhibit the SARS-CoV-2 viral infection through different mechanisms [42].

The present study from Western Colombia focuses on the importance of traditional, cultural, and generational history with reference to the use of important and significant medicinal plants to find a strategy to fight the new virus. According to the use during the COVID-19 treatment process, the following species obtained the highest use values within the various mixtures: *Zingiber officinale*, *Eucalyptus globulus*, *Gliricidia sepium*, *Citrus x limon,* and *Matricaria recutita.* Scientific information has demonstrated their effectiveness in treating these infections [10]. Some of these plant products are promising against respiratory diseases and can be the best source of alternative medicine. Although some clinical shreds of evidence have been reported for some of the compounds, there needs to be an extensive study on the toxicological aspect and interaction with other therapeutics [10,43].

According to the Pasvalys study results, systematized in Table S1, many medical indications of home-made herbal preparations were used without EMA approved indications and were based solely on folk knowledge and experience in medicine and it can be a source of ideas for the further research of ethnic heritage-based medical applications for respiratory system disorders. Herbal formulations regarding medicinal plants used to treat cough (the main indication in our study) may have the potential to alleviate symptoms of long-COVID, whereas plants used for bronchitis and pain may be used in inflammatory symptoms. New possibilities for herbal formulations for respiratory system disorders can be obtained from the analysis of archival data (Table S2), because only 14 of the 69 herbal medicinal raw materials were described in the EMA monographs. Phytochemical studies are also recommended as a large number of phytochemicals have been discovered in many herbs, which may be extracted and used as a primary compound to produce effective antiviral drugs.

## 4. Conclusions

Unlike modern medicines, herbs are often claimed to be non-toxic due to their natural origin and long-term use as traditional medicines. However, numerous difficulties can also occur due to the long-term use toxicity, drug–herb interactions, and misidentification of the plant species. The COVID-19 pandemic can be identified as the cause as it has caused difficulties in reaching a physician, leaving pharmacists as the most widely available health care professionals. On the other hand, the question arises as to whether the consultation and the knowledge of the pharmacist were sufficient and met the expectations of patients. Herbalists are not health care professionals, but the use of herbal home-made preparations during the pandemic was raised in all countries. However, it should be assessed whether the measures recommended by herbalists are in line with the evidence-based indications described in the EMA monographs. Although most indications based on the archival source do not agree with the EMA assessment (Table S2), it may provide useful information for researchers developing herbal pharmaceuticals to alleviate COVID-19-induced conditions. The symptoms caused by COVID-19, especially the troublesome long COVID, are forcing researchers to look for natural products that enhance health and relieve the symptoms. The focus is on the gentle, safe, but effective action of plants for long-term use. The most cited and culturally important plant species *Thymus vulgaris* L., *Tilia cordata* Mill., *Matricaria recutita* L., *Rubus idaeus* L., *Tussilago farfara* L., *Foeniculum vulgare* Mill., *Glycyrrhiza glabra* L., *Salvia officinalis* L., *Mentha x piperita* L., and *Calendula officinalis* L. need further investigation for herbal formulations for the possible treatment of respiratory system disorders as one of the COVID-19 related symptoms. Trends in the ethnopharmacological choices of the modern consumer (Table S1) and the analysis of archival sources (Table S2) can be a great source of ideas for new herbal-based pharmaceutical preparations treating COVID-19 symptoms in Lithuania, emphasizing the EMA guidelines for safe use.

## Figures and Tables

**Table 1:** A comparison with the EMA recommendations.

		Data of the Study	Percent	Archival Data	Percent
EMA assessment	Present	32	53.4%	14	20.3%
	Non present	28	46.6%	55	79.7%
Indications	Correspond to recommendations	16	50%	7	50%
	Not correspond to recommendations	16	50%	7	50%

## Data Availability

The data generated for this study are available from the authors upon request.

## Supplementary Materials

The following supporting information can be downloaded at: https://www.mdpi.com/article/10.3390/ijerph19158905/s1, Table S1: Herbal materials in the Pasvalys district, Lithuania; Table S2: Herbal materials in the archival source, Lithuania; Questionnaire S1.

## References

[1] Wangkheirakpam S. (2018). Traditional and Folk Medicine as a Target for Drug Discovery. Natural Products and Drug Discovery.

[2] Gukauskiene L., Juknyte K. (2019). Lithuanian population attitude to herbal medicine. Int. J. Pharm. Chem. Biol. Sci..

[3] Alarcomicronn R., Pardo-de-Santayana M., Priestley C., Morales R., Heinrich M. (2015). Medicinal and local food plants in the south of Alava (Basque Country, Spain). J. Ethnopharmacol..

[4] Fontefrancesco M., Barstow C., Grazioli F., Lyons H., Mattalia G., Marino M., McKay A.E., Soukand R., Corvo P., Pieroni A. (2019). Keeping or changing? Two different cultural adaptation strategies in the domestic use of home country food plant and herbal ingredients among Albanian and Moroccan migrants in Northwestern Italy. J. Ethnobiol. Ethnomed..

[5] Aprilio K., Wilar G. (2021). Emergence of Ethnomedical COVID-19 Treatment: A Literature Review. Infect. Drug Resist..

[6] Cock I.E., Van Vuuren S.F. (2020). The traditional use of southern African medicinal plants for the treatment of bacterial respiratory diseases: A review of the ethnobotany and scientific evaluations. J. Ethnopharmacol..

[7] Agbor G.A., Ndjib R. (2021). Systematic Review of Plants Used Against Respiratory Diseases Related to COVID-19 in Africa. J. Drug Deliv. Ther..

[8] Franco F.M., Bussmann R.W. (2020). Rising to the occasion: Outlining Ethnobiologists’ response to the coronavirus (COVID-19) pandemic. Ethnobot. Res. Appl..

[9] Vandebroek I., Pieroni A., Stepp J.R., Hanazaki N., Ladio A., Alves R.R.N., Picking D., Delgoda R., Maroyi A., van Andel T. (2020). Reshaping the future of ethnobiology research after the COVID-19 pandemic. Nat. Plants.

[10] Cordoba-Tovar L., Ríos-Geovo V., Largacha-Viveros M.F., Salas-Moreno M., Marrugo-Negrete J.L., Ramos P.A., Chaverra L.M., Jonathan M.P. (2021). Cultural belief and medicinal plants in treating COVID 19 patients of Western Colombia. Acta Ecol. Sin..

[11] Villena-Tejada M., Vera-Ferchau I., Cardona-Rivero A., Zamalloa-Cornejo R., Quispe-Florez M., Frisancho-Triveño Z., Abarca-Meléndez R.C., Alvarez-Sucari S.G., Mejia C.R., Yañez J.A. (2021). Use of medicinal plants for COVID-19 prevention and respiratory symptom treatment during the pandemic in Cusco, Peru: A cross-sectional survey. PLoS ONE.

[12] Khadka D., Dhamala M.K., Li F., Aryal P.C., Magar P.R., Bhatta S., Thakur M.S., Basnet A., Cui D., Shi S. (2021). The use of medicinal plants to prevent COVID-19 in Nepal. J. Ethnobiol. Ethnomed..

[13] Pranskuniene Z., Ratkeviciute K., Simaitiene Z., Pranskunas A., Bernatoniene J. (2019). Ethnobotanical Study of Cultivated Plants in Kaisiadorys District, Lithuania: Possible Trends for New Herbal Based Medicines. Evid. Based. Complement. Alternat Med..

[14] Pranskuniene Z., Dauliute R., Pranskunas A., Bernatoniene J. (2018). Ethnopharmaceutical knowledge in Samogitia region of Lithuania: Where old traditions overlap with modern medicine. J. Ethnobiol. Ethnomed..

[15] Pranskuniene Z., Bernatoniene J., Simaitiene Z., Pranskunas A., Mekas T. (2016). Ethnomedicinal Uses of Honeybee Products in Lithuania: The First Analysis of Archival Sources. Evid. Based Complement. Alternat Med..

[16] Tilvikas J. (2017). Lietuvininkų Liaudies Medicina Nuo XX a. Vidurio iki XXI a. Pradžios. http://www.ekgt.lt/media/dokumentai/veikla/Tyrimai/2017tyrimai/Tilvikas_tyrimas.pdf.

[17] Pieroni A., Vandebroek I., Prakofjewa J., Bussmann R.W., Paniagua-Zambrana N.Y., Maroyi A., Torri L., Zocchi D.M., Dam A.T.K., Khan S.M. (2020). Taming the pandemic? The importance of homemade plant-based foods and beverages as community responses to COVID-19. J. Ethnobiol. Ethnomed..

[18] Alyami H.S., Orabi M.A.A., Aldhabbah F.M., Alturki H.N., Aburas W.I., Alfayez A.I., Alharbi A.S., Almasuood R.A., Alsuhaibani N.A. (2020). Knowledge about COVID-19 and beliefs about and use of herbal products during the COVID-19 pandemic: A cross-sectional study in Saudi Arabia. Saudi Pharm. J..

[19] AnonymousVisuotine Lietuviu Enciklopedija. https://www.vle.lt/straipsnis/taurage/.

[20] Čepaitienė A. (2019). The geolect of Pasvalys: A dialectometric approach to dialectal features. Taikom. Kalbot..

[21] Pasvalys. https://www.pasvalys.lt/lt/savivaldybe/apie-mus/1744.

[22] International Society of Ethnobiology (2006). International Society of Ethnobiology Code of Ethics (with 2008 Additions). http://ethnobiology.net/code-of-ethics/.

[23] European Medicines Agency (2017). Guideline on the Assessment of Clinical Safety and Efficacy in the Preparation of Community Herbal Monographs for Well-Established and of Community Herbal Monographs/Entries to the Community List for Traditional Herbal Medicinal Products/Substances/Preparations. https://www.ema.europa.eu/en/documents/scientific-guideline/guideline-assessment-clinical-safety-efficacypreparation-eu-herbal-monographs-well-established_en.pdf.

[24] WFO 2021: World Flora Online. http://www.worldfloraonline.org.

[25] Stevens P.F. (2012). Angiosperm Phylogeny Website, Version 13. http://www.mobot.org/MOBOT/research/APweb/.

[26] Vilkonis K.K. (2008). Lietuvos Žaliasis Rūbas.

[27] Ragažinskienė O., Rimkienė S., Sasnauskas V. (2005). Vaistinių Augalų Enciklopedija.

[28] Jankevičienė R. (1998). Botanikos Vardų Žodynas.

[29] Luo H., Tang Q., Shang Y., Liang S., Yang M., Robinson N., Liu J. (2020). Can Chinese Medicine Be Used for Prevention of Corona Virus Disease 2019 (COVID-19)? A Review of Historical Classics, Research Evidence and Current Prevention Programs. Chin. J. Integr. Med..

[30] Ekiert H.M., Ramawat K.G., Arora J. (2021). Medicinal Plants. Sustainable Development and Biodiversity.

[31] Soukand R., Kalle R. (2013). Where does the border lie: Locally grown plants used for making tea for recreation and/or healing, 1970s–1990s Estonia. J. Ethnopharmacol..

[32] Pranskuniene Z., Bajoraite R., Simaitiene Z., Bernatoniene J. (2021). Home Gardens as a Source of Medicinal, Herbal and Food Preparations: Modern and Historical Approaches in Lithuania. Appl. Sci..

[33] Soukand R., Quave C.L., Pieroni A., Pardo-de-Santayana M., Tardio J., Kalle R., Luczaj L., Svanberg I., Kolosova V., Aceituno-Mata L. (2013). Plants used for making recreational tea in Europe: A review based on specific research sites. J. Ethnobiol. Ethnomed..

[34] Heinrich M. (2015). Quality and safety of herbal medical products: Regulation and the need for quality assurance along the value chains. Br. J. Clin. Pharmacol..

[35] Šimkūnaitė E. (1948). Lietuvių Liaudies Medicinos Vaistingieji Augalai. Archival material. Available at Lithuanian Museum of the History of Medicine and Pharmacy of the Lithuanian University of Health Sciences.

[36] Ekor M. (2014). The growing use of herbal medicines: Issues relating to adverse reactions and challenges in monitoring safety. Front. Pharmacol..

[37] El Alami A., Fattah A., Chait A. (2020). Medicinal plants used for the prevention purposes during the COVID-19 pandemic in Morocco. J. Anal. Sci. Appl. Biotechnolgy.

[38] Chen S., Dong L., Quan H., Zhou X., Ma J., Xia W., Zhou H., Fu X. (2021). A review of the ethnobotanical value, phytochemistry, pharmacology, toxicity and quality control of *Tussilago farfara* L. (coltsfoot). J. Ethnopharmacol..

[39] Yang Y. (2020). Use of herbal drugs to treat COVID-19 should be with caution. Lancet..

[40] Gupta P.K., Sonewane K., Rajan M. (2021). Scientific rationale of Indian AYUSH ministry advisory for COVID-19 prevention, prophylaxis, and immunomodulation. Adv. Trad. Med..

[41] Mukherjee P.K., Efferth T., Das B., Kar A., Ghosh S., Singha S., Debnath P., Sharma N., Bhardwaj P.K., Haldar P.K. (2022). Role of medicinal plants in inhibiting SARS-CoV-2 and in the management of post-COVID-19 complications. Phytomedicine.

[42] Ang L., Lee H.W., Kim A., Lee J.A., Zhang J., Lee M.S. (2020). Herbal medicine and pattern identification for treating COVID-19: A rapid review of guidelines. Int. Med. Res..

[43] Timalsina D., Pokhrel K.P., Bhusal D. (2021). Pharmacologic Activities of Plant-Derived Natural Products on Respiratory Diseases and Inflammations. BioMed Res. Int..

