# Supplementary material for: Herbal Medicine Uses for Respiratory System Disorders and Possible Trends in New Herbal Medicinal Recipes during COVID-19 in Pasvalys District, Lithuania
Source: Int J Environ Res Public Health. 2022 Jul 22;19(15):8905. doi: 10.3390/ijerph19158905 (PMC9332438; doi:10.3390/ijerph19158905)
Supplement: Supplementary file 1 [file ijerph-19-08905-s001.zip › Table S1. Herbal materials in Pasvalys District, Lithuania.pdf]

Family	*Botanical name*	*Local name*	*Part used*	*Preparation*	*Therapeutic uses (frequency of citations)*	*EVA evaluation*
Acoraceae	*Acorus calamus* L.	*Ajeras*	Rhizomes	Decoction	Cough, pneumonia (1)	
Adoxaceae	*Sambucus nigra* L.	*Šeivamedis*	Flowers	Tea	Cough (4)	EMEA/HMPC/283166/2007<br>(leaves)<br>Cough and cold.<br>EMA/HMPC/44208/2012<br>(fruits)<br>Bronchitis,cough, sinusitis.
Adoxaceae	*Viburnum opulus* L.	*Putinas*	Flowers<br>Fruits	Tea<br>Raw material with honey, warmed-up	Cough (3)<br>Cough (2)	
Apiaceae	*Aegopodium podagraria* L.	*Garšva*	Aerial part	Juice	Respiratory conditions	
Apiaceae	*Anethum graveolens* L.	*Krapai*	Seeds	Tea	Cough (2)	
Apiaceae	*Coriandrum sativum* L.	*Kalendra*	Fruits	Ethanol infusion	Bronchitis (1)	
Apiaceae	*Foeniculum vulgare* Mill.	*Pankolis*	Fruits<br><br>Seeds	Tea<br><br>Tea	Respiratory conditions (5)<br>Secretion in the lungs (1)	
Araliaceae	*Hedera helix* L.	*Gebenė*	Fruits<br>Leaves	Syrup<br>Tea	Cough (1)<br>Secretion in the lungs (5)	EMA/HMPC/586888/2014<br>(leaves)

						Cough facilitator in the presence of a productive cough.
Amaryllidaceae	*Allium cepa* L.	*Svogūnas*	Bulb<br><br><br><br><br>Bulb	Chopped raw material mixed with water and honey (drops to ear)<br>Syrup	Coryza (1)<br><br><br><br><br>Cough (1)	EMA/HMPC/347195/2011<br>(bulb)<br>Cough, bronchitis, asthma, tonsillitis.
Amaryllidaceae	*Allium sativum* L.	*Cibulis*	Bulb<br><br><br>Bulb<br><br>Bulb	Syrup<br><br><br>Raw material with milk<br>Pulp	Lung diseases, bronchitis, asthma (1)<br>Cough (3)<br><br>Sore throat (1)	EMA/HMPC/7685/2013<br>(bulb)<br>Relief of the symptoms of common cold.
Betulaceae	*Betula pubescens* Ehrh.	*Raistaberžis*	Buds<br>Bark	Tea<br>Decoction	Cough (4)<br>Respiratory conditions, cancer (1)	
Brassicaceae	*Armoracia rusticana* L.	*Krienas*	Roots<br><br><br>Roots<br><br><br>Roots	Grated raw material with lemon juice<br>Grated raw material with lemon juice<br>Grated raw material with lemon juice	Bronchitis, asthma (2)<br><br>Bronchitis (1)<br><br><br>Chronical coryza (1)	
Brassicaceae	*Brassica nigra* L.	*Garstyčia*	Leaves	Compress	Cough (1)	
Boraginaceae	*Borago officinalis* L.	*Agurklė*	Leaves<br><br>Aerial part	Decoction with honey<br>Decoction	Cough (1)<br><br>Cough (1)	

Boraginaceae	*Pulmonaria officinalis* L.	*Vaistinė plautė*	Aerial part<br>Aerial part	Tea<br>Tea	Cough (3)<br>Respiratory conditions (2)	
Boraginaceae	*Symphytum officinale* L.	*Agurkinė, taukažolė*	Roots	Ethanol infusion	Cough (1)	EMA/HMPC/572846/2009<br>(roots)<br>Sprains and bruises.
Campanulaceae	*Lobelia inflata* L.	*Lobelija*	Flowers<br>Leaves	Tea<br>Tea	Cough (1)<br>Cough (1)	
Caprifoliaceae	*Valeriana officinalis* L.	*Valerijonas*	Roots	Ethanol infusion	Cough (1)	EMA/HMPC/150848/2015<br>(roots)<br>Sleep disorders andtemporary insomnia; Mental stress and mood disorders
Compositae	*Achillea millefolium* L.	*Kraujučiai*	Flowers	Tea	Asthma, (immunomodulator) (1)	EMA/HMPC/143949/2010<br>(flowers)<br>Bloating and flatulence, Small superficial wounds,<br>Loss of appetite
Compositae	*Arctium lappa* L.	*Varnalėša*	Flowers<br>Seeds<br>Leaves<br>Roots	Tea<br>Tea<br>Compress<br>Ethanol infusion	Cough (1)<br>Cough (1)<br>Cough (1)<br>Tonsillitis (1)	EMA/HMPC/246763/2009<br>(roots)<br>Seborrhoeic skin conditions,<br>Loss of appetite,<br>Adjuvant in minor urinary complaints
Compositae	*Artemisia abrotanum* L.	*Diemedis*	Leaves<br>Leaves	Tea<br>Ethanol infusion	Tonsillitis (1)<br>Tonsillitis (1)	

Compositae	*Calendula officinalis* L.	*Medetkos*	Flowers	Tea	Respiratory conditions (6)	EMA/HMPC/437450/2017 (flowers)<br>Skin inflamations and minor wounds;<br>Minor inflamations in the mouth ot the throat
Compositae	*Cichorium intybus* L.	*Cikorija*	Flowers<br>Roots	Tea<br>Tea	Cough (2)<br>Bronchus relaxing (1)	
Compositae	*Inula helenium* L.	*Debesylas*	Rhizomes with roots	Decoction	Cough, respiratory conditions (1)	
Compositae	*Matricaria recutita* L.	*Ramunėlės*	Flowers	Tea	Cough (reduces bronchospasm, relaxes muscles) (19)	EMA/HMPC/55843/2011 (flowers)<br>Cough and cold,<br>Minor ulcers and inflamations of the mouth nand throat/ irritations of skin and mucosae
Compositae	*Tussilago farfara* L.	*Šalpusnis*	Flowers<br>Flowers<br><br>Leaves<br>Leaves<br><br>Leaves	Tea<br>Decoction<br><br>Tea<br>Decoction<br><br>Syrup	Cough (5)<br>Lung diseases (1)<br>Cough (5)<br>Sore throat (1), lung diseases (1)<br>Respiratory conditions, asthma(1)	
Cupressaceae	*Juniperus communis* L.	*Kadagys*	Fruits<br>Fruits	Tea<br>Raw material	Cough (1)<br>Cough (1)	EMA/HMPC/441929/2008 (fruits)

						Adjuvant in minor urinary complaints,<br>Digestive disorders (dyspepsia, flatulence)
Equisetaceae	*Equisetum arvense* L.	*Asiūklis*	Aerial part	Tea	Cough (4)	EMA/HMPC/278091/2015<br>(aerial part)<br>Adjuvant in minor urinary complaints<br>Superficial wounds
Fabaceae	*Glycyrrhiza glabra* L.	*Saldymedis (saldmedis)*	Leaves	Tea	Cough (5),<br>irritated mucous membrane (1)	EMA/HMPC/571119/2010<br>(roots)<br>Cough and cold
Fabaceae	*Trifolium pratense* L.	*Dobilas*	Flowers	Ethanol infusion	Cough (2)	
Geraniaceae	*Pelargonium odoratissimum* L.	*Jeronimas*	Leaves	Tea	Cough (5),<br>sore throat,for rinsing (1)	
Grossulariaceae	*Ribes nigrum* L.	*Juodieji*	Leaves	Tea	Cough (1)	EMA/HMPC/745353/2016<br>(leaves)<br>Articular pain<br>Adjuvant in minor urinary complaints
Hypericaceae	*Hypericum perfoliatum* L.	*Jonažolė*	Aerial part	Tea	Respiratory conditions (2)	EMA/HMPC/7695/2021<br>(aerial part)<br>Depressive disorders<br>Mental exhaustion<br>Skin inflamation and minor wounds<br>Gastrointestinal discomfort

Juglandaceae	*Juglans regia* L.	*Graikinis riešutas*	Leaves	Ethanol infusion	Tuberculosis (and throat tuberculosis) (1)	EMA/HMPC/346737/2011<br>(leaves)<br>Skin inflamations
Lamiaceae	*Glechoma hederacea* L.	*Šliaužiančioji traumažolė*	Flowers<br><br>Seeds	Decoction<br><br>Decoction	Liquefies the secretion (1)<br>Liquefies the secretion (1)	
Lamiaceae	*Hyssopus officinalis* L.	*Juozažolė*	Aerial part	Decoction	Asthma (1)	
Lamiaceae	*Lavandula angustifolia* Mill.	*Levanda*	Flowers	Inhalation	Sore throat, Cough (1)	EMA/HMPC/734125/2010<br>(flowers)<br>Mental stress and sleep disorders<br>Mental exhaustion
Lamiaceae	*Melissa officinalis* L.	*Melisa (bitžolė)*	Leaves	Inhalation	Cough (1)	EMA/HMPC/196745/2012<br>(leaves)<br>Mental stress and sleep disorders<br>Mental exhaustion.<br>Digestive disorders (dyspepsia, flatulence)
Lamiaceae	*Mentha aquatica* L.	*Vandens mėta*	Leaves	Tea	Cough (1)	
Lamiaceae	*Mentha x piperita* L.	*Mėta (šaltmėtė)*	Leaves<br><br>Leaves<br>Leaves<br>Leaves	Ethanol infusion<br>Tea<br>Inhalation<br>Inhalation	Cough (1)<br><br>Cough (1)<br>Cough (4)<br>Rhinitis (1)	EMA/HMPC/572705/2014<br>(leaves)<br>Digestive disorders (dyspepsia, flatulence)
Lamiaceae	*Origanum vulgare* L.	*Raudonėlis (raudonėlė)*	Aerial part	Tea	Cough (2)	

Lamiaceae	*Salvia officinalis* L.	*Šalavijas (šalavija)*	Leaves<br>Leaves<br>Leaves	Tea<br>Tea<br>Compress	Tuberculosis (3)<br>Cough (2)<br>Tuberculosis (1)	EMA/HMPC/277152/2015<br>(leaves)<br>Mouth/throat inflamation
Lamiaceae	*Thymus vulgaris* L.	*Čiobreliai*	Flowers	Tea	Cough (23)	EMA/HMPC/342332/2013<br>(aerial part)<br>Cough and cold
Malvaceae	*Alcea rosea* L.	*Aukštoji piliarožė*	Flowers	Tea	Cough (2)	
Malvaceae	*Althaea officinalis* L.	*Vaistinė svilarožė*	Roots<br><br>Roots	Ethanol infusion<br>Ethanol infusion	Cough (2)<br><br>Irritated mucous membrane (1)	EMA/HMPC/436679/2015<br>(roots)<br>Oral or pharyngeal irritation and associated dry cough
Malvaceae	*Tilia cordata* Mill.	*Liepa*	Flowers	Tea	Cough (19)	EMA/HMPC/337066/2011<br>(flowers)<br>Common cold<br>Mental stress
Myrtaceae	*Eucalyptus globulus* Labill.	*Eukaliptas*	Leaves	Inhalation	Cough (1), irritated mucous membrane (1)	EMA/HMPC/892618/2011<br>(leaves)<br>Cough
Pinaceae	*Picea abies* L.	*Eglė*	Needles	Ethanol infusion	Cough (1)	
Pinaceae	*Pinus sylvestris* L.	*Pušis*	Buds<br>Buds	Tea<br>Decoction	Cough (4)<br>Cough (1)	
Plantaginaceae	*Plantago major* L.	*Gyslotis (trauklapis)*	Leaves	Tea	Cough (bronchitis) (3)	EMA/HMPC/437858/2010 (leaves)

						Oral or pharyngeal irritation and associated dry cough
Rosaceae	*Fragaria vesca* L.	*Žemuogė*	Leaves	Tea	Cough (1)	EMA/HMPC/432278/2015<br>(leaves)<br>Adjuvant in minor urinary complaints<br>Diarrhoea
Rosaceae	*Potentilla erecta* L.	*Miškinė sidabražolė*	Roots<br><br>Roots<br>Rhizomes	Ethanol infusion<br>Tea<br>Decoction	Respiratory conditions (3)<br>Cough (1)<br>Tonsillitis (1)	EMA/HMPC/5513/2010<br>(roots)<br>Inflamation of the oral mucosa<br>Diarrhoea
Rosaceae	*Potentilla recta* L.	*Stačioji sidabražolė*	Roots	Decoction	Lung cancer (1)	
Rosaceae	*Prunus padus* L.	*Ieva*	Flowers<br>Leaves<br>Fruits<br>Bark	Tea<br>Tea<br>Tea<br>Decoction	Cough (1)<br>Cough (1)<br>Cough (1)<br>Tonsillitis (1)	
Rosaceae	*Rubus idaeus* L.	*Avietės*	Aerial part with roots	Tea	Sore throat (14)<br>After lung surgery (1)	EMA/HMPC/44211/2012<br>(leaves)<br>Minor inflamation of the mouth and throat
Scrophulariaceae	*Verbascum densiflorum* Bertol.	*Tūbė*	Flowers<br><br>Leaves	Ethanol infusion<br>Ethanol infusion	Cough (2)<br><br>Cough (2)	EMA/HMPC/611537/2016<br>(flowers)<br>Oral or pharyngeal irritation and associated dry cough

Solanaceae	*Solanum dulcamara* L.	*Karklavijas*	Stem<br><br>Leaves	Decoction<br><br>Tea	Cough, asthma (1)<br>Cough (1)	EMA/HMPC/734361/2011<br>(stem)<br>Recurrent eczema
Urticaceae	*Urtica dioica* L.	*Dilgynė*	Leaves<br><br>Roots	Decoction with honey<br>Decoction with honey	Astma (2)<br><br>Cough (1)	EMA/HMPC/508015/2007<br>(leaves)<br>Articular pains<br>Adjuvant in minor urinary complaints<br><br>EMA/HMPC/461160/2008<br>(roots)<br>Lower urinary tract symptoms related to benign prostatic hyperplasia
Zingiberaceae	*Zingiber officinale* Roscoe	*Imbieras*	Roots	Tea	Cough (3)	EMA/HMPC/749154/2010<br>(rhizome)<br>Gastrointestinal disorders (bloating and flatulence)
Xanthorrhoeaceae	*Aloe arborescens* Mill.	*Alijošius*	Leaves<br><br><br><br>Leaves	Pulp<br><br><br><br>Juice	Cough, chronic bronchitis, pneumonia, tuberculosis (1)<br>Cough, bronchitis (4)	
