# Supplementary material for: Herbal Medicine Uses for Respiratory System Disorders and Possible Trends in New Herbal Medicinal Recipes during COVID-19 in Pasvalys District, Lithuania
Source: Int J Environ Res Public Health. 2022 Jul 22;19(15):8905. doi: 10.3390/ijerph19158905 (PMC9332438; doi:10.3390/ijerph19158905)
Supplement: Supplementary file 1 [file ijerph-19-08905-s001.zip › Table S2. Herbal materials in the archival source, Lithuania.pdf]

Family	***Botanical name***	***Local name***	***Part used***	***Preparation***	***Administration and usage***	***EVA evaluation***
Adoxaceae	*Viburnum opulus* L.	*Putinas*	Fruits<br>Fruits<br><br>Fruits	Ethanol infusion<br>Diluted infusion<br><br>Ethanol infusion, mixture with water (equally)	Throat disorders<br>Throat problems, for rinsing<br>Compress	
Amaryllidaceae	*Allium ursinum* L.	*Česnakas*	Bulb	Raw material	Cough, Tuberculosis	
Apiaceae	*Chaerophyllum aromaticum L.*	*Gurgždis*	Aerial parts<br><br>Seeds	Decoction with milk and honey<br>Decoction	Sore throat, coryza<br>Cough	
Apiaceae	*Daucus carota* L.	*Morka*	Aerial parts with roots	Decoction	Cough	
Borraginaceae	*Anchusa officinalis* L.	*Karvės liežuvis*	Roots<br>Aerial parts	Decoction<br>Decoction	Chest pain, cough<br>Chest pain, cough	
Borraginaceae	*Borrago officinalis* L.	*Agurkažolė*	Flowers<br><br>Leaves<br><br><br>Aerial parts with roots	Decoction<br><br>The leaves are crushed with warm, freshly milked milk<br>Decoction	Bronchitis, dry cough<br>Chest disorders<br><br><br>Constant pains	
Borraginaceae	*Cynoglossum officinale* L.	*Šunlielė*	Aerial parts with roots	Decoction (with water or milk)	Cough, chest pain, throat conditions	
Borraginaceae	*Symphytum officinale* L.	*Živakotas, kaulė*	Flowers	Decoction	For throat rinsing	EMA/HMPC/572846/2009<br>(roots)

						Sprains and bruises
Brassicaceae	*Armoracia rusticana L.*	*Krienas*	Roots<br><br>Roots<br><br>Roots	Ethanol infusion<br><br>Compress<br><br>Grated roots	Dyspnoea, pneumonia, corryza<br>Dyspnoea, pneumonia, corryza<br>Dyspnoea, pneumonia, corryza	
Campanulaceae	*Jasione Montana* L.	*Rutulėliai, medinukai*	Flowers	Decoction with milk	Cough, throat hoarseness, tuberculosis	
Campanulaceae	*Lobelia inflata* L.	*Lobelija*	Aerial parts	For smoking	Dyspnoea	
Cannabaceae	*Humulus lupulus* L.	*Apinys*	Aerial parts	Decoction with honey	Coughing attacks	EMEA/HMPC/513617/2006<br>(strobiles)<br>Mental stress and sleep disorders
Caprifoliaceae	*Succisa pratensis* M.	*Miegalė*	Flowers	Decoction	Cough, throat conditions	
Caryophyllaceae	*Saponaria officinalis* L.	*Putoklis*	Flowers	Decoction (1-2 pinch for 1 liter water)	Coughing attacks	
Compositae	*Calendula officinalis* L.	*Medetka*	Flowers	Decoction with milk and honey	Bronchitis, dry cough, bronchitis, respiratory diseases	EMA/HMPC/437450/2017<br>(flowers)<br>Skin inflamations and minor wounds;<br>Minor inflamations in the mouth ot the throat
Compositae	*Centaurea cyanus* L.	*Rugiagėlė, vosylkos*	Flowers	Decoction	Cough	
Compositae	*Centaurea jacea* L.	*Bajorė*	Flowers	Decoction	Cough	

Compositae	*Eupatorium cannabinum* L.	*Kameras, kanapininkas*	Aerial parts	Decoction with milk and honey	Cough, tuberculosis	
Compositae	*Inula helenium* L.	*Debesylas*	Flowers	Tuberculosis	Tuberculosis	
Compositae	*Lactuca sativa* L.	*Salota*	Flowers	Decoction	Heavy cough	
Compositae	*Senecio Jacobaea* L.	*Jokūbinė žilė*	Flowers	Decoction	Heavy cough	
Compositae	*Senecio palustris* L.	*Pilkaplautė žilė*	Flowers	Decoction	Heavy cough	
Compositae	*Tanacetum vulgare* L.	*Ežė, čižmas*	Aerial parts with roots	Decoction	For throat rinsing	
Compositae	*Taraxacum officinale* L.	*Pienė, pabradė*	Flowers	Decoction	Cough, tuberculosis	EMA/HMPC/475726/2020 (roots with herb Mild digestive disorders (abdominal fullness, flatulence, slow digestion); Loss of apetite; Adjuvant in minor urinary complaints
Compositae	*Tussilago farfara* L	*Močiakalapis*	Flowers<br>Leaves	Decoction<br>Decoction	Cough, tuberculosis<br>Cough, cold	
Ephedraceae	*Ephedra distachya* L.	*Asiūklakrūmis*	Aerial parts	Decoction	Dyspnoea	
Fabaceae	*Melilotus albus* Medik.	*Jondobilis*	Aerial parts	Decoction with milk	Cough, hoarseness, chest pain	
Fabaceae	*Melilotus officinalis* L.	*Barkūnas*	Aerial parts	Decoction (with milk or honey)	Cough, hoarseness, respiratory conditions, chest pain	EMA/HMPC/44166/2016 (aerial part)

						Heaviness in the legs (venous circulatory disorders), minor skin inflammation
Fabaceae	*Trifolium repens* L.	*Dobiliukai*	Flowers	Decoction with milk	Throat and lung diseases	
Lamiaceae	*Ajuga genevensis* L.	*Stačioji vaisgina*	Aerial parts	Decoction	Cold, cough,	
Lamiaceae	*Lavandula spica* L.	*Lavandra*	Flowers	Decoction with milk or with diluted honey	Cough,	
Lamiaceae	*Majorana hortensis* M.	*Mairūnas*	Aerial parts	Ethanol infusion	Heavy cough, dyspnoea, lung diseases	
Lamiaceae	*Mentha arvensis* L.	*Mėta*	Leaves<br><br>Leaves	Decoction<br><br>Hot sweetened decoction	Sore throat, cough,<br>Cold	
Lamiaceae	*Mentha crispa* L.	*Mėta garbanota*	Leaves<br>Leaves	Decoction<br>Diluted decoction	Cough, sore throat<br>For rinsing, gargle, compress	
Lamiaceae	*Stachys officinalis* L.	*Vaistinė notra*	Aerial parts	Decoction	Dry cough, whooping-cough, dyspnoea	
Lamiaceae	*Stachys paluster* L.	*Pelkinė notra*	Aerial parts	Decoction	Cold,<br>cough	
Lamiaceae	*Stachys silvatica* L.	*Miškinė notra*	Aerial parts	Decoction	Cold,<br>cough	
Lamiaceae	*Thymus serpyllum* L.	*Marijos*	Flowers<br><br>Flowers	Decoction<br><br>Diluted decoction	Cold<br>cough, chest pain<br>For washing, rinsing, compress	

Malvaceae	*Althaea officinalis* L.	*Piliarožė*	Roots	Decoction	Cough	
Malvaceae	*Malva alcea* L.	*Dedešva kiauliarožė*	Flowers	Tea	Cough, throat conditions	
Malvaceae	*Malva naglecta* Wallr.	*Paprastoji dedešva*	Flowers	Decoction	Cough, hoarseness, chest pain	EMA/HMPC/749510/2016<br>(leaves)<br>Oral and pharyngeal irritation and associated dry cough;<br>Gastrointestinal discomfort
Malvaceae	*Malva sylvestris* L.	*Rožiukė*	Flowers	Decoction	Cough, hoarseness	EMA/HMPC/749510/2016<br>(leaves)<br>Oral and pharyngeal irritation and associated dry cough;<br>Gastrointestinal discomfort
Malvaceae	*Mlva verticillata* L.	*Rauktalapė dedešva*	Flowers<br>Flowers	Decoction<br>Decoction	Cough, hoarseness.<br>Washing, rinsing at hoarseness	
Papaveraceae	*Chelidonium majus* L.	*Ugniažolė*	Aerial parts	Decoction	Cough	
Papaveraceae	*Papaver argemone* L.	*Aguona*	Petals/ Buds	Decoction	Heavy cough	
Papaveraceae	*Papaver dubium* L.	*Aguona*	Half riped fruits/ Flowers	Decoction	Cough, colic in the lungs	

Papaveraceae	*Papaver somniferum* L.	*Aguona*	Seeds<br><br>Petals	Ripe seeded milk (sweetened)<br>The petals are boiled with honey to make a mash and drunk with hot milk	Cough, chest pain<br><br>Cough	
Pinaceae	*Pinus sylvestris* L.	*Pušis*	Resin<br><br>Young shoots/ Buds<br><br>Young shoots	Turpentine<br><br>Ethanol infusion; decoction (with milk or with honey)<br>The shoots are crushed to a porridge and diluted with warm milk, filtered, honey added	Cough, cold, pneumonia<br>Tuberculosis<br><br>Tuberculosis	
Poaceae	*Brachypodium sylvaticum* P.Beauv.	*Strugė*	All plant with roots	Decoction	Heavy cough	
Poaceae	*Elytrigia repens* (L.) Nevski	*Vėmukas, vėjas*	Rhizome	Tea	Heavy cough, dyspnoea	
Poaceae	*Hordeum vulgare* L.	*Ketureilis*	Germinated seeds<br><br>Seeds<br><br>Young greens	Decoction<br><br>Ethanol infusion of sprouted grains<br>The greens are rubbed with honey, poured with hot milk	Heavy cough, chest pain, „dry pain“<br>Heavy cough<br><br>Tuberculosis	

Poaceae	*Triticum vulgare* Vill.	*Kvietys*	Unthreshed spica	Decoction	Cold;<br>to induce sweating	
Primulaceae	*Anagallis arvensis* L.	*Progailis*	Aerial parts	Ethanol infusion	Heavy cough	
Primulaceae	*Primula veris L.*	*Raktažolė*	Flowers<br>All plant with roots	Decoction<br>Decoction	Cold, dry cough,<br>Throat tuberculosis dyspnoea	EMEA/HMPC/64684/2007<br>(flowers)<br>Expectorant in cough associated with cold
Ranunculaceae	*Aquilegia vulgaris* L.	*Sinavadas*	All plant with roots<br>All plant with roots	Decoction with honey<br>Ethanol infusion	Cough, dyspnoea<br><br>Cough, dyspnoea	
Rosaceae	*Alchemilla vulgaris* L.	*Našlaičių ašarėlės, Marijos ašarėlės*	Aerial parts	Decoction	Cough, „blood spitting“	
Rosaceae	*Filipendula ulmaria* L.	*Vingiris*	Aerial parts / Flowers	Decoction	Cough, cold	
Rosaceae	*Rosa centifolia* L.	*Rožė*	Flowers	Decoction	Cough, hoarseness	EMA/HMPC/137299/2013<br>(flowers)<br>Skin inflamation;<br>Inflamation in the oral and pharyngeal mucosa
Rosaceae	*Sorbus aucuparia* L.	*Šermukšnis*	Flowers<br>Unriped fruits/ Leaves	Decoction<br>Decoction /Tea	Cough<br>Pneumonia	
Rubiaceae	*Asperula odorata* L.	*Miglelė*	Aerial parts	Decoction	Respiratory diseases,<br>cough, dyspnoea,<br>throat tuberculosis	

Rubiaceae	*Galium Mollugo* L.	*Lipikas*	Flowers<br><br><br>Flowers	Decoction with milk<br><br>Decoction	Heavy cough, bronchitis, dyspnoea<br>Respiratory diseases	
Sapindaceae	*Aesculus hippocastanum* L.	*Kaštonas*	Flowers<br>Pericarp<br>Fruits<br>Bark	Decoction<br>Decoction<br>Roasted fruit tea<br>Decoction	Cough<br>Whooping -cough<br>Heavy cough<br>Cough	EMA/HMPC/354156/2011<br>(bark)<br>Chronic venous insufficiency;<br>Venous circulatory disturbances;<br>Bruises of oedema and haematoma<br>EMEA/HMPC/225319/2008<br>(seeds)<br>Chronic venous insufficiency;<br>Venous circulatory disturbances;<br>Bruises of oedema and haematoma
Schisandraceae	*Illicium verum* Hook.f.	*Marijos žvaigždė, kiniškos žvaigždės*	Fruits<br>Fruits	Ethanol infusion<br>Crushed fruit with honey	Chest pain<br>Cough	
Scrophulariaceae	*Verbascum nigrum* L.	*Meškos ausis*	Truss<br><br>Truss, leaves	Decoction<br><br>Decoction	Respiratory diseases<br>Dyspnoea	
Scrophulariaceae	*Verbascum thapsus* L.	*Gaurė*	Truss<br><br>Truss	Decoction<br><br>Diluted decoction	Cough, dyspnoea, chest pain<br>For rinsing and gargle	EMA/HMPC/611537/2016<br>(flowers)

						Sore throat associated with dry cough and cold
Solanaceae	*Solanum dulcamara* L.	*Velniuogė*	Aerial parts with roots<br>Flowers/fruits	Decoction<br><br>Decoction	Cough, dyspnoea<br><br>Respiratory diseases	EMA/HMPC/734361/2011<br>(stem)<br>Recurrent eczema
Theaceae	*Camellia sinensis* L.	*Arbatmedis*	Leaves	For smoking	Dyspnoea	EMA/HMPC/283630/2012<br>(leaves)<br>Fatigue and weakness
Violaceae	*Viola arenaria* DC.	*Žibutė, fiolka,žibikutė*	Roots	Ethanol infusion	Dyspnoea, cough	
Xanthorrhoeaceae	*Aloe vera* L.	*Alijošius*	Leaves<br><br>Leaves	Leaf pulp with honey<br>The cut leaves are fermented with honey for two weeks, then mixed with milk	Heavy cough, tuberculosis<br>Tuberculosis	
